# Combination of phototherapy with immune checkpoint blockade: Theory and practice in cancer

**DOI:** 10.3389/fimmu.2022.955920

**Published:** 2022-09-02

**Authors:** Yujie Zhao, Xu Liu, Xinyu Liu, Jing Yu, Xin Bai, Xi Wu, Xinyu Guo, Zhihui Liu, Xiaowei Liu

**Affiliations:** ^1^Laboratory of Integrative Medicine, Clinical Research Center for Breast, State Key Laboratory of Biotherapy, West China Hospital, Sichuan University, Chengdu, China; ^2^Department of Head, Neck and Mammary Gland Oncology, Cancer Center, West China Hospital, Sichuan University, Chengdu, China

**Keywords:** immune checkpoint blockade, photothermal therapy (PTT), photodynamic therapy (PDT), immunogenic cell death (ICD), tumor-infiltrating T cells, nanoparticles

## Abstract

Immune checkpoint blockade (ICB) therapy has evolved as a revolutionized therapeutic modality to eradicate tumor cells by releasing the brake of the antitumor immune response. However, only a subset of patients could benefit from ICB treatment currently. Phototherapy usually includes photothermal therapy (PTT) and photodynamic therapy (PDT). PTT exerts a local therapeutic effect by using photothermal agents to generate heat upon laser irradiation. PDT utilizes irradiated photosensitizers with a laser to produce reactive oxygen species to kill the target cells. Both PTT and PDT can induce immunogenic cell death in tumors to activate antigen-presenting cells and promote T cell infiltration. Therefore, combining ICB treatment with PTT/PDT can enhance the antitumor immune response and prevent tumor metastases and recurrence. In this review, we summarized the mechanism of phototherapy in cancer immunotherapy and discussed the recent advances in the development of phototherapy combined with ICB therapy to treat malignant tumors. Moreover, we also outlined the significant progress of phototherapy combined with targeted therapy or chemotherapy to improve ICB in preclinical and clinical studies. Finally, we analyzed the current challenges of this novel combination treatment regimen. We believe that the next-generation technology breakthrough in cancer treatment may come from this combinational win-win strategy of photoimmunotherapy.

## Introduction

Over the past several years, with the recognition of tumor immune escape, several immune checkpoint molecules have been identified for cancer immunotherapy, such as PD-1: PD-L1 and CTLA-4: CD80/CD86 (1–3). Tumors escape those T cell based immune surveillance by crippling T cells’ functionality *via* upregulating the expression of immune checkpoint molecules (4). Blocking the interaction of PD-1 with PD-L1 or CTLA4 with CD80/CD86 restores T cell function, restarting and amplifying the antitumor immune response. Thus, immune checkpoint inhibitors (ICIs) against CTLA4 and PD-1 and its ligand PD-L1, such as Ipilimumab, Pembrolizumab, Nivolumab, and Atezolizumab, have been used to treat the malignant tumors and significantly improve the survival of patients (5–8). Immune checkpoint blockade (ICB) therapy has become a routine treatment for more than 20 different indications (9–11), including non-small cell lung cancer (12, 13), colon cancer (14, 15), esophageal cancer (16, 17), melanoma (18), renal cell carcinoma (19), bladder or urothelial carcinoma (20), breast cancer (21).

Although ICB has held great promise for cancer treatment, the efficacy of ICB only benefits a minority of patients, which is due to the low response rates (22). For example, only approximately 20% of non-small cell lung cancer patients respond to ICB therapy (23). Moreover, the response rate of ICB alone is less than 40% in melanoma (24), less than 20% in hepatocellular carcinoma (25), and less than 10% in triple-negative breast cancer (26). This low therapeutic response rate accounts for many tumors that have evolved sophisticated strategies to evade immune surveillance. Generally speaking, there are four main reasons for a poor response to ICB: 1) tumor antigen deficiency, 2) insufficient infiltration of T lymphocytes, 3) defects in the tumor antigen processing and presentation mechanism, and 4) an immunosuppressive tumor microenvironment (TME) (27). In conclusion, insufficient T cell infiltration caused by the lack of tumor-specific antigens is the main reason for the failure of ICB therapy.

In recent years, phototherapy, especially nanoparticle-based photoimmunotherapy, has been recognized as an effective strategy for promoting T cell infiltration and improving the efficiency of ICB. Nanoparticle-based phototherapy, mainly including photothermal therapy (PTT) and photodynamic therapy (PDT), exhibits potent antitumor efficacy, minimal invasiveness, slight side effects, and immune regulator functions in tumor treatment (28, 29). During PTT, photothermal agents convert light energy into heat under near-infrared (NIR) light irradiation to kill tumor cells (30). PDT destroys tumor cells by exogenously reactive oxygen species (ROS) generated from light irradiated photosensitizers (31). In cancer treatment, both PTT and PDT can induce the immunogenic cell death (ICD) of tumor cells. ICD is characterized by the release of damage-associated molecular patterns (DAMPs), tumor-associated antigens (TAAs), neoantigens, and proinflammatory cytokines from dying tumor cells that activate tumor-specific immune responses, including activating immune effector cells, promoting T -cells infiltration, and enhancing the secretion of cytokines (32). In addition, photothermal and photodynamic agents can also be coloaded with immunostimulant, small molecular inhibitors, chemotherapeutic drugs, or immunoadjuvants for effective combination treatments. For example, albumin paclitaxel has been loaded into gold nanocage nanoparticles for enhanced antitumor efficiency and ICD levels (33). Importantly, PTT and PDT have been approved clinically by numerous regulatory agents to treat local tumors, such as melanoma, esophageal cancer, and lung cancer (34, 35). Based on the advantage of phototherapy in regulating antitumor immune responses and promoting T cell infiltration, phototherapy combined with ICB is considered a promising strategy for cancer treatment. Indeed, PTT and PDT have been combined with ICIs for the treatment of malignant tumors and metastatic tumors in preclinical and clinical settings, especially for those patients who lack tumor-infiltrating T cells.

**Figure 1:**
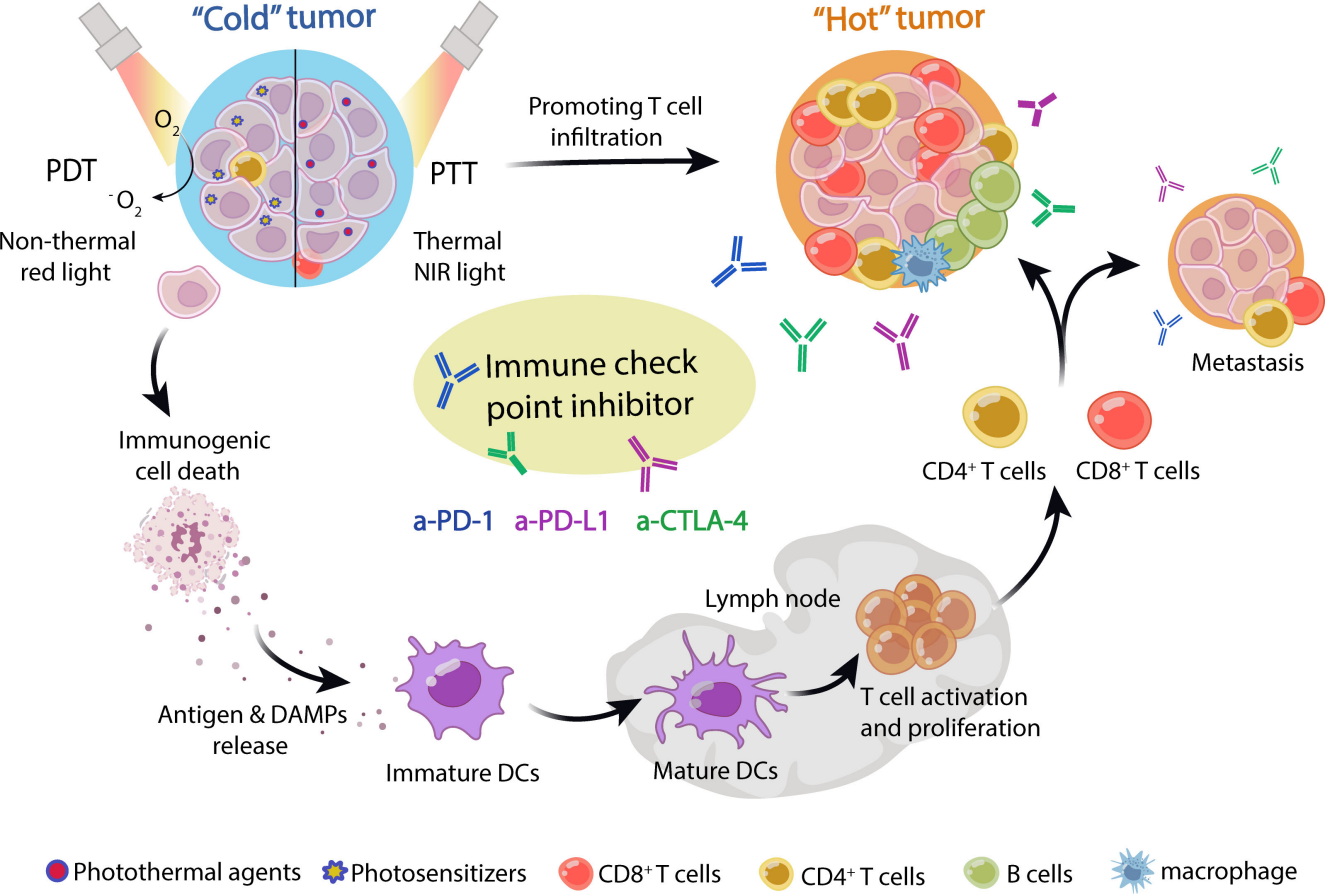
Schematic of phototherapy combined with ICB in cancer treatment. Nanoparticle-loaded photosensitizers or photothermal agents were efficiently enriched in tumor tissue. Upon laser irradiation, photosensitizers or photothermal agents generate reactive oxygen species (ROS) and heat energy in PTT and PDT, respectively, which induces immunogenic cell death (ICD) of tumors. The dying tumor cells released tumor neoantigens and DAMPs, which stimulated DC maturation and activated T cells. Then, the tumor-specific T cells were recruited into tumor niches and activated by ICIs, including anti-PD-1, anti-PD-L1, and anti-CTLA-4 antibody, which could effectively treat primary and metastatic tumors, as well as immune “cold” tumors. Thus, phototherapy can synergize with immune checkpoint blockade to treat tumors.

Above all, combining nanoparticle-based phototherapy and ICB therapy can improve the treatment outcome and prevent tumor recurrence and metastasis by activating a specific antitumor immune response (**Figure 1**). Moreover, nanoparticle-based phototherapy is a promising strategy to coordinate the combination of ICB with other treatment strategies, such as targeted therapy and chemotherapy. Here, we summarized the mechanism of phototherapy in cancer immunotherapy and discussed the current progress of phototherapy combined with ICB therapy to treat malignant tumors, including melanoma, non-small cell lung cancer, esophageal cancer, etc., in pre-clinical and clinical settings. In addition, the progress of phototherapy combined with targeted therapy or chemotherapy to improve ICB has also been summarized. Finally, the current challenges, deficiencies, and future improvements of this novel combination treatment regimen have also been analyzed.

## Phototherapy for cancer treatment and immune regulation

Phototherapies, including PTT and PDT, have been used to treat malignant tumors in the clinic for more than 40 years (35). Several tumor types, including melanoma, esophageal, lung tumors, hepatocellular carcinoma, glioma, etc., have shown good response rates to PTT and PDT (36). In addition to providing an elegant solution to ablate primary tumors, phototherapy also provides a localized source of tumor antigens and DAMPs, promoting antigen presentation and increasing systemic immunity to prevent tumor progression and metastasis. In fact, different types of phototherapies have different mechanisms of action: PTT can induce thermal tissue damage and achieve tumor thermal ablation without damaging normal cells and tissues, while PDT can induce chemical damage by generating ROS based on the local activation of photosensitizers in tumors. Therefore, in this section, we discuss the different mechanisms for anticancer immunotherapy treatment in PTT and PDT.

### Role of PTT in cancer immunotherapy

**Figure 2:**
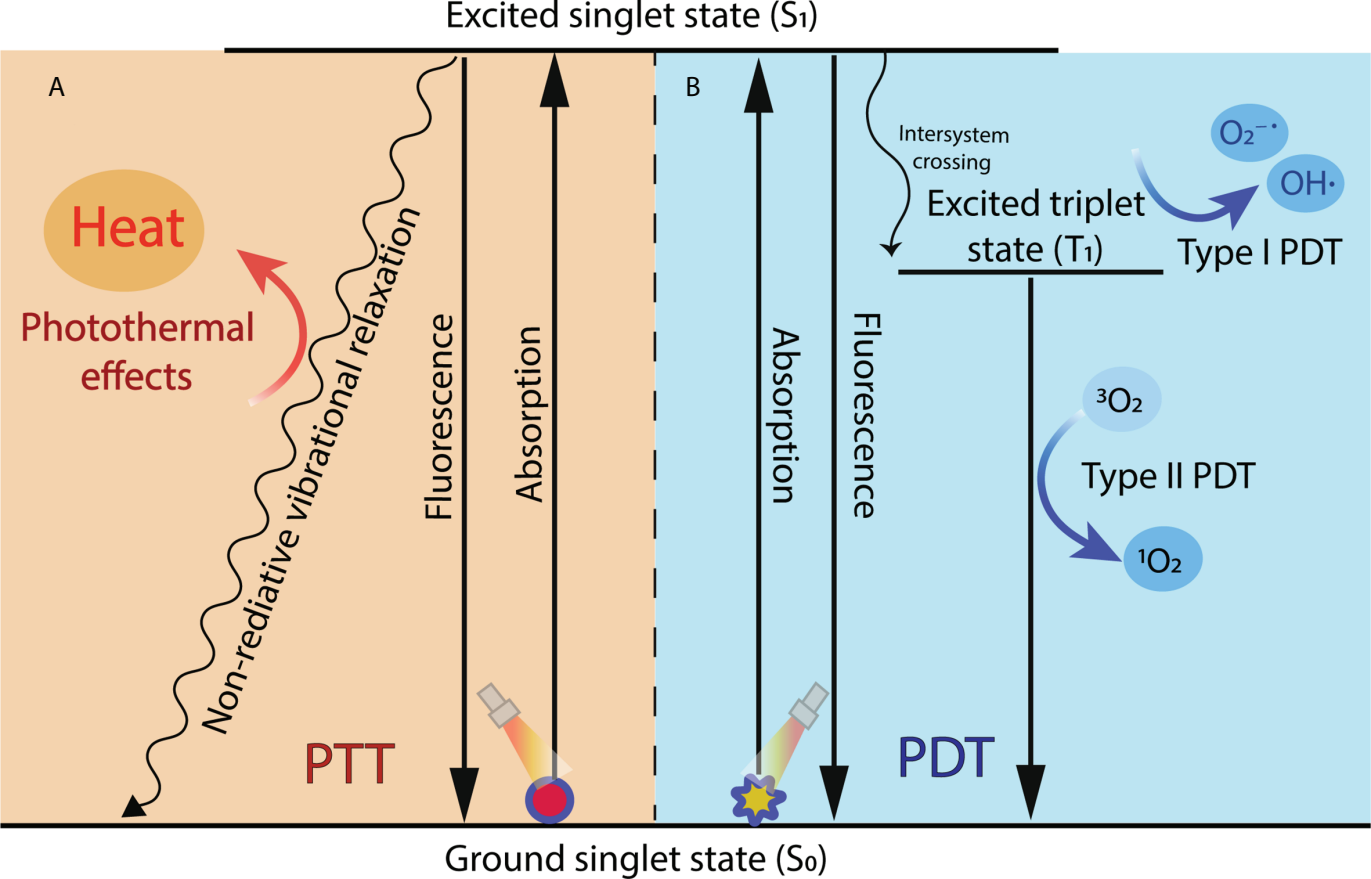
Mechanisms of photodynamic reaction and photothermal effect during PDT and PTT. **(A)** In PTT, when excited singlet state photothermal agents return to the ground state, they undergo nonradiative vibrational relaxation, increasing the surrounding microenvironment temperature and causing cell death. **(B)** In PDT, the excited singlet state of the photosensitizer transforms to a more stable excited T_1_ through intersystem crossing and then finally generates ROS through type I reaction or type II reaction.

PTT can take advantage of the photothermal effects of photothermal agents, obtaining energy from laser irradiation and converting it into heat. The absorption wavelength of photothermal agents is mostly in NIR-I window (750-1000 nm). At present, A small number of photothermal agents of NIR-II (1000-1500 nm) have been exploited. NIR-II light has better biological tissue penetration than red light (620-750 nm) and NIR-light (37). Once irradiated by light of a specific wavelength, the PTT agents absorb photon energy, migrating from the ground singlet state (S_0_) to an excited singlet state (S_1_). As the S_1_ state PTT agents return to the S_0_ state, they must undergo vibrational relaxation, a nonradiative form of decay that results in collisions between the excited photothermal agents and their surrounding molecules (38). This increased kinetic energy increases the surrounding microenvironment temperature (**Figure 2A**). When the temperature reaches 41°C, changes will happen in gene expression patterns, such as the generation of heat-shock proteins (HSP), to mitigate the effects of the initial thermal damage (39). Mild hyperthermia approximately 41-42°C can promote increased blood flow and improve the tumor’s vascular permeability, enhancing the delivery of anticancer drugs to tumors (40). A further high temperature can cause tumor vessels to collapse, undergo necrosis, apoptosis, coagulation, and hypoxia. When the temperature of tumor tissue rises to 42°C, irreversible tissue damage will occur (41). For example, 42-46°C for 10 min will result in cell necrosis. In particularly, cell death is rapidly accelerated at 46-52°C owing to microvascular thrombosis and ischemia. When the temperature is above 60°C, protein denaturation happens, and the plasma membrane melts, leading to almost instant cell death (35).

In addition to directly killing tumor cells *via* hyperthermia, PTT also induces ICD in tumor cells, which releases TAAs and DAMPs to trigger the adaptive immune response and activate the tumor-specific immune response (42). Remarkably, PTT can provoke anticancer vaccine effects and produce long-term antitumor efficacy *in vivo*. DAMPs include high-mobility group box-1 (HMGB1), calreticulin (CRT), HSP90, HSP70, and adenosine triphosphate (ATP) (32). When thermal damage occurs, HMGB1 and ATP, serving as a “find me” signal for recruiting antigen-presenting cells (APCs), are released to the outside of the cells. CRT exposed on the cell membrane surface promotes phagocytosis by APCs as an “eat-me” signal (43). Moreover, HSPs can bind to TAAs to form HSP-antigen complexes, which can effectively activate APCs. Activated APCs migrate to lymphoid organs, where tumor antigens are presented to T cells, initiating a T cell-mediated immune response to eradicate cancer cells (44). These characteristics of PTT make it the ideal candidate for combined with immunotherapy. Photothermal immunotherapy is a new concept that combines PTT with immunotherapy. It can generate synergistic thermal immune effects, enhancing the control of primary tumors and metastases. ICD induced by PTT has been developed in combination with immunotherapies such as ICIs, immune adjuvants, and CAR T cell therapy to directly eliminate tumors and induce sustained antitumor immune effects. At present, photothermal immunotherapy has evolved from a concept to a promising clinical treatment for metastatic cancer.

### Role of PDT in cancer immunotherapy

Unlike the direct tumor cell killing of PTT, PDT induces tumor cell apoptosis and necrosis by ROS generated from photosensitizers. PDT relies on photochemical reactions among three nontoxic components: photosensitizers, light, and oxygen dissolved in the cells. In general, photosensitizers used in PDT can be classified into three generations based on their evolution. The first generation of photosensitizers was constituted by naturally occurring porphyrins and their derivatives. Their phototherapy performance was limited by their red-light wavelength (about 630 nm) of excitation, which is difficult to reach deep tumors (45). The second-generation photosensitizers are synthetic compounds primarily based on porphyrins and chlorine structures. Compared with the first generation, the second-generation photosensitizers have more high purity, photosensitivity, tissue selectivity, and longer absorption wavelength in the visible-NIR (650-800 nm) (46). However, they still had poor water-solubility, body clearance rate, and low tumor selectivity. In recent years, third-generation photosensitizers are developed to solve the disadvantage of the second by utilizing chemical modification, nano-delivery system, or antibody conjugation (47). Another important trigger of PDT is laser irradiation. Upon laser irradiation, the electronic state of the photosensitizer is converted from the singlet primary energy state (S_0_) to the unstable excited singlet state (S_1_). Subsequently, part of the energy of the photosensitizer is radiated in the form of a quantum of fluorescence, transforming to a more stable excited triplet state (T_1_). In addition, the T_1_ state generates ROS through two mechanisms: type I and type II photodynamic reactions (48). In the type I pathway, T_1_ directly reacts with endogenous cancerous substrates, such as cell membranes or biological macromolecules, and produces free radicals and anion radicals through a hydrogen or electron transfer. The radicals may further react with O_2_ and water to generate ROS, including superoxide anions (O_2_^−^˙) and hydroxyl radicals (OH˙). In the type II pathway, T_1_ transforms the basic energetic state (the basic triplet state) O_2_ into highly cytotoxic reactive singlet oxygen (^1^O_2_) by direct energy transfer. The generated singlet oxygen can oxidize macromolecular cellular components, such as nucleic acids, lipids, and proteins, which leads to cellular death by either apoptosis or necrosis (48–50) (**Figure 2B**).

PDT can kill tumor cells by generating ROS and activating an immune response against tumor cells. The different intracellular locations of photosensitizers will cause different types of ICD. For example, apoptosis is caused by mitochondria, the cell membrane destruction induces necrosis, and autophagy is provoked by lysosomes/endoplasmic reticulum damage (49). An acute inflammatory reaction and neoplasm infiltration by leukocytes can be led by local injuries and oxidative stress in tumor tissue induced by different types of PDT. In the tumor cells, damaged endothelial cells, and tumor stromal cells, PDT caused a rapid and massive release of proinflammatory mediators and cytokines, which participate in the recruitment of neutrophils and other myeloid cells. These proinflammatory mediators include arachidonic acid, cytokines, histamine, and the complement system. The cytokines include tumor necrosis factor, interleukin (IL)-1β, and IL-6. PDT also induces tumor ICD, which leads to the activation of antitumor immunity through danger signaling mechanisms caused by the activation of DAMPs. This process stimulates innate immunity, resulting in adaptive immune responses (51). In addition, ROS damage to vascular endothelial cells can activate clotting processes. Persistent tumor tissue hypoxia caused by the aggregation of platelets and the blockage of blood vessels leads to cell death (49). These properties of PDT support the combination of PDT with immunotherapy. Photodynamic immunotherapy has been used clinically in combination with immune stimulators and ICIs.

## Mechanism and preclinical progress on phototherapy combined with ICB

**Figure 3:**
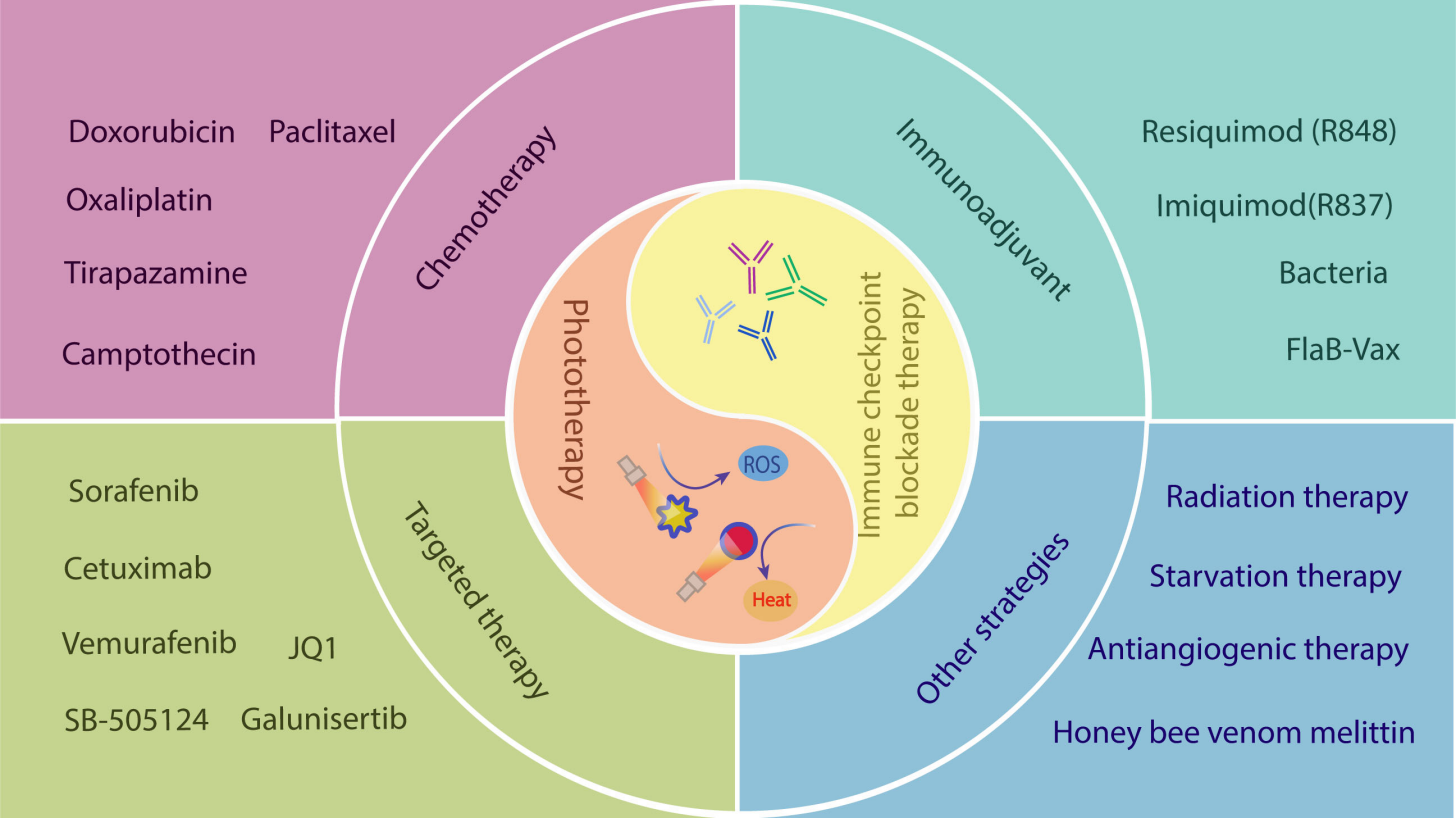
PTT/PDT combined with other treatment strategies to improve ICB therapy. PTT and PDT could be combined with different therapeutic modalities, including targeted therapy, chemotherapy, immune adjuvant therapy, starvation therapy, and antiangiogenic therapy to improve the antitumor immune response of ICB and inhibit tumor growth, metastasis, and recurrence.

ICIs exert excellent antitumor effects by restoring the cytotoxic function of tumor-specific T cells. However, infiltrating T cell is the prerequisite for effective anti-CTLA-4/PD-1 therapy, and not all tumors express ligands that bind to ICIs. For multiple cancers, the response rate of ICB only ranges from 10% to 40%, resulting in a large proportion of patients not benefiting from ICI treatment (52). In contrast, PTT or PDT can kill tumor cells through noninvasive apoptosis or ablation, promoting T cell infiltration into the tumor tissue. They have been introduced to T cell-based ICB therapy to enhance the systemic antitumor immune response. In addition, many current preclinical studies on multidrug combination therapy platforms have demonstrated the combinational treatment’s potent inhibition of tumor growth, metastasis, and recurrence. Current approaches include ICB combined with PTT/PDT or further combined with targeted therapy, chemotherapy, and immune adjuvants based on ICB+PTT/PDT (**Figure 3**). However, the immune responses induced by PTT and PDT are complicated *in vivo*, and the exact molecular mechanism is not fully understood. Therefore, in this section, we will discuss the signaling pathway regulated by phototherapy and review the progress in preclinical research on the combination of ICB and phototherapy.

### The signaling pathway regulated by phototherapy and ICB

**Figure 4:**
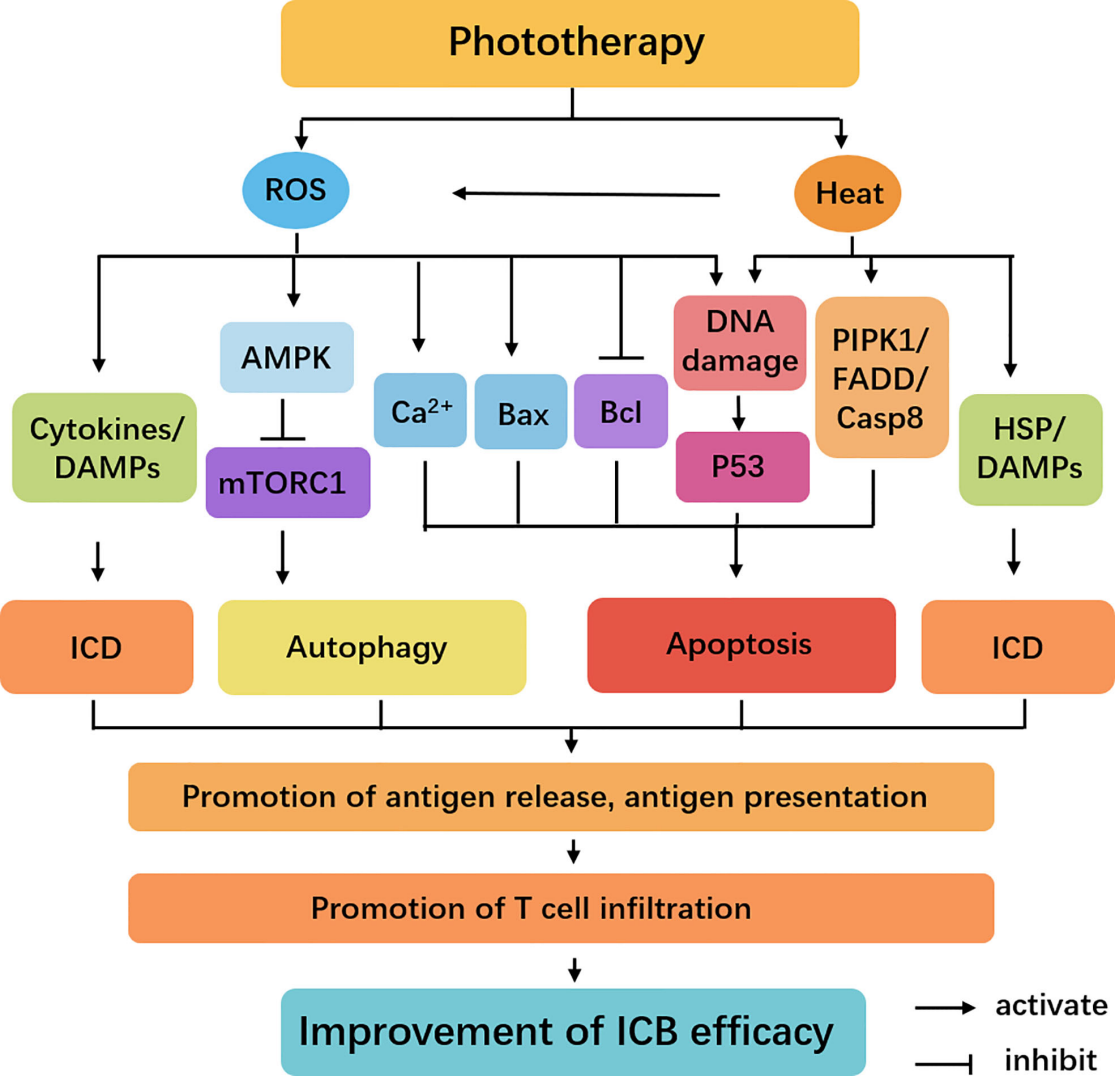
Cellular signaling pathways regulated by phototherapy and ICB. The irradiation triggers the activation of heat and ROS-mediated AMPK, Bax, Bcl, p53, and RIPK1 signaling. These pathways have different roles in the induction of apoptotic cell death and protective autophagy. Meanwhile, several DAMPs and cytokines play important roles in ICD. These dead cancer cells release antigens, promoting the tumor antigen processing and presentation and T cell infiltration to improve the efficiency of ICB.

Besides inducing ICD of tumoral cells by releasing DAMPs to trigger the maturation of DCs and activation of CD8^+^ T cells, phototherapy also regulates a variety of intracellular signal transduction pathways through generating ROS and heat (**Figure 4**). High temperatures can induce receptor interacting protein kinase (RIPK)-1, fas-associated death domain (FADD). And the upregulated RIPK1 and FADD in tumor cells can associate with caspase-8 to induce apoptosis (53–56). ROS promotes activation of death receptor pathways by inhibiting the production of Bcl-2/Bcl-xL and upregulating the expression of apoptotic-related proteins Bax. Meanwhile, the increased levels of ER stress and disruption of Ca^2+^ homeostasis induce intrinsic apoptosis (57). Generated heat and ROS by phototherapy can cause DNA damage, which leads to p53 activation (58). p53 is a tumor suppressor transcription factor that is known for its pro-apoptotic activity. Recently, an intriguing study demonstrates that PDT-induced p53 can re-educate tumor-associated macrophages (TAMs) to M1 antitumor phenotype, thereby stimulating antitumor T cell activation. p53 was positively correlated with M1-like macrophage makers (TNFα and IL1β) but was negatively correlated with M2-like macrophage markers (Arg1 and CCL16). In addition, p53 level was positively correlated with immune cells infiltration levels, including that B cells, CD8^+^ T cells, CD4^+^ T cells, macrophages, neutrophils, and DCs. More interestingly, they discovered laser irradiation could induce p53 expression in a dose-dependent manner, providing new insight into phototherapy enhanced antitumor immune response (59). In addition, ROS-induced energy stress can strongly activate AMPK, and AMPK can upregulate autophagy by inhibiting mTORC1 activity (60). Therefore, phototherapy not only directly kills tumor cells but also improves the efficiency of ICIs by regulating the tumor immune environment.

### Phototherapy improves the efficiency of ICB therapy

Given that phototherapy has proven to regulate antitumor immune response, the combination of PTT/PDT and ICB therapy has developed into a novel treatment regimen for cancer therapy. PTT/PDT selectively destroys cancer cells and induces ICD, initiating the local immune reaction by releasing antigens from dying cancer cells. Thus, combining phototherapy with ICB therapy may improve antitumor efficacy.

During the past years, PTT has been introduced to ICB to magnify the systemic antitumor immune response and applied to treat multiple cancers (**Table 1**) (99). For example, Ye and coworkers coated black phosphorus quantum dots with the surgically removed 4T1 tumor cell membrane to construct black phosphorus quantum dot nanovesicles (BPQD-CCNVs) as a kind of personalized photothermal vaccine (70). Under 808 nm laser irradiation, BPQD generates heat energy, which promotes GM-CSF and LPS continuously released from the nanoparticle. The released GM-CSF and LPS recruit and stimulate DCs, which activate the tumor-specific T cells to kill tumor cells. In addition, the rising temperature also upregulated the expression of co-stimulatory molecules, such as CD80, CD86, MHC-I, and MHC-II molecules, further enhancing the ability to cross-present antigens and stimulate T cells. Moreover, administration of PD-1 antibody together with BPQD-CCNVs nanovesicles significantly promote CD8^+^ T cell infiltration and eliminate surgical residual and lung metastatic tumors. This study demonstrates that photosensitive nanovesicles can be developed into individualized tumor vaccines and synergize with immunotherapy. Another work by Ran and coworkers reported a copper sulfide (CuS)-based nano-platform combined with PD-1 antibodies to obliterate primary tumors and inhibit metastatic tumors (71). Some photothermal agents, such as single-walled carbon nanotube (SWNT) and Prussian Blue (PB), can act as immunological adjuvants to promote the maturation of DCs and the production of antitumor cytokines (67). For example, in 2016, Fernandes et al. described a strategy of PBNP-based PTT with anti-CTLA-4 checkpoint inhibition for treating neuroblastoma (68). When combined with ICB, this photothermal agent with an immune-boosting effect could significantly inhibit metastatic tumor proliferation.

**Table 1:** Preclinical studies of phototherapy-ICB combined therapy or plus other therapeutics.

Phototherapy	Phototherapy agent	ICB	Combinationtherapy	Combinationtherapy agent	Cancermodel	Ref.
PDT	IRDye700	PD-1	alone	alone	4T1 Breast cancer	(61)
PDT	ZnP@pyro	PD-L1	alone	alone	4T1 Breast cancer	(62)
PDT	Fe-TBP	PD-L1	alone	alone	CT26 Colon cancer	(63)
PDT	ZnPc	CTLA-4	alone	alone	4T1 Breast cancer	(64)
PDT	PcN4	PD-L1	alone	alone	4T1 Breast cancer	(65)
PDT	Porphyrin	PD-L1	alone	alone	4T1 Breast cancer	(66)
PTT	SWNTs	CTLA-4	alone	alone	4T1 Breast cancer	(67)
PTT	PBNP	CTLA-4	alone	alone	Neuro2a Neuroblastoma	(68)
PTT	GNPs	PD-L1	alone	alone	HCC827 Lung cancer	(69)
PTT	BPQDs	PD-1	alone	alone	4T1 Breast cancer	(70)
PTT	CuS	PD-1	alone	alone	MDA-MB-231 Breast cancer	(71)
PDT	IRDye800CW	PD-L1	targeted therapy	cetuximab	CT26 Colon cancer	(72)
PTT	IR780	PD-1	targeted therapy	SB-505124	4T1 Breast cancer	(73)
PTT	AuNC	PD-1	targeted therapy	Vemurafenib	SMM103 Melanoma	(74)
PTT	PDMNs	PD-L1	targeted therapy	JQ1	4T1 Breast cancer	(75)
PTT	AuNC	PD-L1	targeted therapy	galunisertib	CT26 Colon cancer	(76)
PTT	PBNP	PD-L1	targeted therapy	sorafenib	HepG2 Hepatoma	(77)
PDT	Ce6	PD-L1	chemotherapy	paclitaxel	4T1 Breast cancer	(78)
PDT	Ce6	PD-1	chemotherapy	doxorubicin	4T1 Breast cancer	(79)
PDT	Pyrolipid	PD-L1	chemotherapy	oxaliplatin	MC38/CT26 Colon cancer	(80)
PDT	Ce6	PD-L1	chemotherapy	doxorubicin	4T1 Breast cancer	(81)
PDT	UCNPs-MOFs	PD-L1	chemotherapy	tirapazamine	CT26 Colon cancer	(82)
PDT	Ce6	PD-L1	chemotherapy	doxorubicin	4T1 Breast cancer	(83)
PDT	AuNp	PD-L1	chemotherapy	doxorubicin	CT26 Colon cancer	(84)
PTT	IRDye 800	PD-L1	chemotherapy	camptothecin	4T1 Breast cancer	(85)
PTT	Pd NP	PD-L1	chemotherapy	doxorubicin	CT26 Colon cancer	(42)
PTT	IR780	PD-L1	chemotherapy	oxaliplatin	CT26 Colon cancer	(86)
PTT	Ag prism	PD-1	chemotherapy	doxorubicin	4T1 Breast cancer	(87)
PDT	Ce6	CTLA-4	immune adjuvant	R837	4T1 Breast cancer	(88)
PDT	W-TBP	PD-L1	immune adjuvant	CpG	TUBO Breast cancer	(89)
PDT	PhA	PD-1	immune adjuvant	FlaB-Vax	B16-F10 Melanoma	(90)
PDT	UCNPs-Ce6	CTLA-4	immune adjuvant	R837	CT26 Colon cancer	(91)
PTT	HAuNS	PD-1	immune adjuvant	CpG	CT26 Colon cancer	(92)
PTT	Fe_3_O_4_ NPs	PD-L1	immune adjuvant	R837	4T1 Breast cancer	(93)
PTT	ICG	CTLA-4	immune adjuvant	R837	CT26 Colon cancer	(94)
PTT	CDs	PD-L1	immune adjuvant	R848	4T1 Breast cancer	(95)
PDT	IRDye700	PD-L1	antiangiogenic therapy	anti-CD276	4T1 Breast cancer	(96)
PDT	Ce6	PD-1	honey bee venom melittin	MLT peptide	4T1 Breast cancer	(97)
PTT+PDT	Cu_2_MoS_4_	CTLA-4	starvation therapy	GOx	U14 Cervical cancer	(98)

PDT can induce ICD by releasing CRT exposure and dying tumor cell debris, resulting in the activation of T cells to kill the residual tumor. Recently, Dr. Liu and his colleagues demonstrated that PDT synergistically promotes the antitumor efficacy of ICI (61). They developed a phthalocyanine dye-labeled probe to mediate PDT, significantly inhibiting tumor growth and T cells infiltration in a 4T1 mouse model. Based on PDT, PD-1 blockade also inhibited lung metastasis formation by agitating a systematic antitumor immune response. A ZnP@pyro PDT treatment was reported to sensitize tumors to PD-L1 antibody in a similar work from Lin et al. Their work also eradicated the primary 4T1 breast tumor and significantly prevented metastasis in the lung (62). Another physiologically self-degradable microneedle-assisted platform for combining PDT and anti-CTLA4 antibody can generate similar synergistic reinforcement outcomes while can reduce the side effects of treating breast cancer (64). Furthermore, since PDT can induce enhancement of hypoxia, Yoon et al. utilized the photosensitizer phthalocyanine derivatives (PcN4) to deliver hypoxia-activated prodrug (AQ4N), and create a more hypoxic TME for the activation of AQ4N. When combined with ICB therapy, it enables efficient abscopal responses and enhances antimetastatic effects in breast cancer treatment (65).

Downregulating the *de novo* expression of PD-L1 may be another new research direction for ICB therapy. Interestingly, nanomaterials of PTT can be used as gene silence carriers for PD-L1 due to their excellent modified performance. In the work of Cui and coworkers, they designed a new nanoplatform based on gold nanoprisms (GNPs) to carry PD-L1 siRNA. The platform not only functioned as a carrier for siRNA delivery to downregulate the PD-L1 expression but also served as the photothermal agent for PTT (69). In another work, Chen et al. synthesized a type of cationic flexible organic framework nanoparticle loaded with porphyrin of PDT and siRNA to mediate PD-L1 gene silencing to achieve an excellent antitumor effect, providing a basis for developing nanophotosensitizers and excellent gene carriers (66). In combination with ICB therapy, the nanomedicine of PTT and PDT not only achieved a superior effect in suppressing the growth of the primary tumor but also promoted long-lasting immune memory to inhibit tumor recurrence and metastasis.

### Phototherapy combined with targeted therapy to improve ICB therapy

Targeted therapies inhibit the growth and progression of cancer cells by interfering with specific targeted molecules (100). Small-molecule inhibitors or monoclonal antibodies of targeted therapy, such as inhibitors for epidermal growth factor receptor (EGFR) (101), transforming growth factor (TGF)-β (102), BRAF (103), VEGF (104), and MYC (105), can produce impressive tumor responses in selected patients while having potentially fewer side effects. These inhibitors not only directly kill tumor cells but also activate the immune system through a variety of mechanisms, such as promoting tumor antigen processing and presentation, increasing intratumoral T cell infiltration, enhancing T cell function, and attenuating the immunosuppressive effect of the tumor microenvironment (106). For example, kinase inhibitor vemurafenib can enhance the induction of MHC Class I and Class II molecules by IFN-γ and IFNα2b, thereby enhancing antigen presentation and promoting antitumor immune response (107, 108). Since many of the targeted therapeutic agents can directly or indirectly modulate immune cell functions, combining ICB with targeted therapy has become a promising therapeutic strategy for malignant tumors. However, targeted drugs have some limitations: 1) poor aqueous solubility, 2) inhibition of normal cells that share targeted kinases, and 3) low bioavailability. Nanoparticle-based phototherapy loaded with targeted drugs can avoid these disadvantages. Moreover, this multidrug combination therapy can realize the dual advantages of PTT/PDT and targeted therapy, which is more conducive to immunotherapy (**Table 1**).

### EGFR signaling pathway

EGFR, a transmembrane receptor, is one of the four closely related receptor tyrosine kinases and is involved in regulating cellular multiplication, survival, differentiation, metastasis, and plays a crucial role in the occurrence and immune escape of various malignant tumors. EGFR has historically served as the primary target for treating uncontrolled colorectal cancer growth. For example, Jin et al. reported a novel cerasome nanoparticle decorated with cetuximab, an anti-EGFR antibody, IRDye800CW, and MRI contrast DOTA-Gd to enable *in vivo* tumor detection and PDT. The nanoparticles they designed possess significant potential for the dual-modality imaging-guided precise PDT of colorectal cancer due to its high ability to target tumors. Combining EGFR-targeted PDT with PD-L1 immunotherapy could achieve superior therapeutic efficacy without tumor recurrence (72).

### TGF-β signaling pathway

TGF-β signal is involved in the proliferation, differentiation, adhesion, movement, and metabolism of tissue cells. Dysregulation of the TGF-β signaling pathway in TME is closely related to the blocking of T cell differentiation, the production of Treg subsets, and the restrained tumor-killing effect of CTLs, thus leading to a protumor immune environment (109). Suppose the TGF-β inhibitor is loaded into the nanosystem to specify delivery to tumor tissues. In that case, the inhibitor can reduce the generation of Treg cells and simultaneously avoid severe adverse reactions caused by nonselective systemic delivery. Recently, Li et al. coloaded TGF-β inhibitor (SB-505124) and photosensitizer (IR780) into nanoliposomes. PTT induced primary tumor ICD, allowing more CTL to infiltrate the tumor tissues and reducing Treg cells when incorporated with TGF-β inhibitor. In this work, PTT combined with PD-1/PD-L1 checkpoint blockade further unleashes T cells to attack 4T1 tumor cells (73). Yan et al. synthesized gold nanocages called GNC-Gal@CMaP functionalized with macrophage membrane and anti-PD-L1 antibody. They used GNC-Gal@CMaP load with galunisertib to improve synergistic PTT and immunotherapy against colorectal cancer (76). The nanocomposites designed can be selectively accumulated in the tumors, eliminate the primary tumor mass, and inhibit distant tumor growth *via* the abscopal effect.

### MAPK signaling pathway

MAPK belongs to a large family of serine-threonine kinases, forming major cell-proliferation signaling pathways from the cell surface to the nucleus. The Ras/Raf/MAPK (MEK)/ERK pathway is the most critical signaling cascade among MAPK signal pathways (110). It plays an essential role in the survival and development of tumor cells. However, most patients treated with MAPK pathway inhibitors do not respond well to PD-1 immunotherapy (111). Using PTT/PDT nanomaterials can avoid this problem of insufficient tumor antigen presentation after MAPK pathway inhibitors resistance. A recent study by our team found that MAPK-targeted therapy impeded antitumor Tcell signatures in the tumor relapse phase by attenuating HSPs mediated antigen presentation. To address this problem, we developed a gold nanoparticle to load MAPK pathway inhibitor. Nanoparticle-mediated PTT and MAPK pathway targeted therapy can efficiently inhibit tumor cell growth and promote HSP expression, which promotes T cell infiltration and enhances the antitumor immune response in melanoma treatment. Based on PTT and MAPK pathway targeted therapy, PD-1 immunotherapy can efficiently convert immune “cold” tumors into “hot” ones and inhibit tumor growth. Our study revealed that gold particle mediated PTT can coordinate targeted therapy and ICB therapy, which provided a novel strategy for treating multifocal tumors and immune “cold” tumors (74).

### Other signaling pathways

Currently, multikinase inhibitors that can simultaneously target more than one pathway have been approved for cancer treatment. Sorafenib (SF) is the first systemic therapy approved for hepatocellular carcinoma. It is a protein kinase inhibitor with activity against many protein kinases, including VEGFR, PDGFR, and RAF kinases (112). In recent work, Tian and colleagues designed hepatocellular carcinoma-targeted nanoparticles conjugated PB to load SF and combined with an anti-PD-L1 antibody to treat hepatocellular carcinoma. The combination treatment strategy effectively eliminates tumor cells at the primary site by nanoparticle-mediated SF targeted inhibition of photothermal effects. The antitumor effects produced by local treatment can be extended to the whole body and enable the establishment of long-term immunological memory, inhibiting tumor metastasis and recurrence (77). For another example, Lu et al. designed polydopamine nanoparticles (PDMNs) encapsulating JQ1 to treat triple-negative breast cancer. The JQ1-loaded PDMNs accumulated in the tumor tissue and released JQ1 in a self-degradable manner. The released JQ1 inhibits the growth of triple-negative breast cancer by inhibiting the BRD4-c-MYC axis and suppressing the expression of PD-L1, which facilitates the activation of T cells in tumor tissue. Meanwhile, PDMN transforms light energy into heat and ablates tumors upon laser irradiation (75).

### Phototherapy combined with chemotherapy to improve ICB therapy

For many tumor types, chemotherapy still represents the therapy of choice. Some studies have shown that chemotherapy drugs such as doxorubicin (Dox), oxaliplatin (Oxa), and paclitaxel (PTX) can induce tumor cell death in an immunogenic manner, playing a synergistic antitumor effect with immunotherapy (113). However, the problems of low targeting, systemic toxicity, and poor water solubility of chemotherapy drugs are unavoidable. Nanoparticles of PTT/PDT can not only be loaded with chemotherapy drugs to achieve targeted delivery but also be combined with chemotherapy to induce ICD and facilitate antitumor ICB immune therapy. Many studies have used nanotechnology to carry out triple phototherapy, chemotherapy, and immunotherapy treatments (**Table 1**) (80–82).

Oxa, a chemotherapeutic drug approved by FDA for the treatment of colorectal cancer, induces cell death by triggering apoptosis and stimulating CRT exposure (114). Lin and colleagues reported using nanoscale coordination polymer core-shell nanoparticles to carry Oxa in the core and a photosensitizer-lipid conjugate in the shell for effective chemotherapy and PDT to treat colon cancer. The result showed that chemotherapy and PDT synergized with ICB have the highest response rate compared to all controls in a bilateral colon carcinoma tumor model, with even induction of abscopal effects and induced cell death in distant tumors that were not irradiated (80). In another example, a tumor-targeting thermosensitive liposomal system carrying PD-L1 inhibitors, IR780, and Oxa, promoted antigen presentation and lymphocyte infiltration to enhance colon cancer immunotherapy (86). Their work proved that chemotherapy/PDT provides an efficient way to induce immunogenicity in the TME and enhance the antitumor immunity of anti-PD-L1 antibodies.

Dox-mediated ICD therapy has considerable potential in cancer treatment. However, undesirable drug delivery efficiency and unavoidable toxicity limit the ICD efficiency of Dox (115). Photothermal sensitive nanoparticles can avoid systemic toxicity and improve the ICD efficiency of chemotherapy. For example, Yang et al. developed a cascade chemo-photodynamic therapy (chemo-PDT) by loading Dox and Chlorine E6 (Ce6) into ROS-sensitive lipid-polymer hybrid nanoparticles. Under 660 nm laser irradiation, ROS were generated by the encapsulated Ce6, which works for cancer treatment and enhances intracellular DOX release. Based on this cascade combo regimen, administrating the PD-L1 antibody could efficiently inhibit primary tumor growth and ablate distant tumors (81). In addition, similar works are MMP2-responsive controlled-release systems for colon cancer therapy (42) and silver nano prisms core-shell nanoplatforms for breast cancer treatment (87). Combinational strategies like these can enhance the antitumor responses of ICIs against both primary and distant tumors.

PTX is a first-line anticancer chemotherapy agent. It can enhance immunotherapy efficacy by reversing immunosuppression in the TME (116). Combined PDT with PTX can improve the effects of a-PD-L1 even for treating tumors with low immunogenicity (117). Recently, Zhang et al. developed a chemo-PDT to enhance the therapeutic effect of PD-L1 immunotherapy by loading photosensitizer Ce6 and PTX into mesoporous silica nuclear nanoparticles. Nanoparticle-mediated chemo-PDT can induce the antitumor immune response and improve the therapeutic effect of PD-L1 blockade in primary and metastatic tumors (78). Camptothecin (CPT) possesses the most effective cytotoxicity for tumor cells in the S and G2 phases. For example, a polypyrrole-loaded CPT-conjugated HA nanoparticle (P@CH) was developed for tumor targeting and PDT combinational therapy. When combined with anti-PD-L1 immunotherapy, the primary tumor was completely depleted, and the lung metastasis were not observed. The result was much better than anti-PD-L1 antibody treatment, indicating that the combination could enhance the immunotherapy of ICIs (85).

### Phototherapy combined with immunoadjuvants to improve ICB therapy

PTT and PDT are highly immunogenic treatments with the potential to recruit DCs to the TME by releasing tumor cell debris and TAAs. However, within the local TME, there are a variety of inhibitory immune cells and molecules, which are unfavorable for cancer immune treatment of combined PTT/PDT and ICB. Another strategy for enhancing immune reactions is exposure of DCs to toll-like receptor (TLR) agonists, such as imiquimod (R837), resiquimod (R848), and CpG. At present, some studies have reported loading PTT/PDT nanocarriers with immunoadjuvants and then combining them with ICB to induce a robust systemic antitumor immune response (**Table 1**).

R837 is a TLR-7 agonist that can promote DCs to phagocytize TAAs and mature, enhancing the activation and proliferation of antigen-specific lymphocytes in draining lymph nodes (118). Liu and his colleagues designed a light-triggered *in situ* gelation system containing a photosensitizer (88). Immune adjuvant R837 was further introduced into this system to trigger robust antitumor immune responses after PDT. With the help of the immune adjuvant, the hydrogel system could significantly enhance immune responses by multiround stimulation. Further combined with CTLA4 blockade offered the abscopal effect to inhibit the growth of distant tumors. It provided adequate long-term immune memory protection from a rechallenged tumor. Another multifunctional UCNP-based platform coloaded Ce6 and R837 onto polymer-coated UCNPs to treat colorectal cancer (91). The presence of those R837-containing nanoparticles as the adjuvant can promote strong antitumor immune responses. Efficiency was further promoted by the anti-CTLA-4 blockade to effectively eliminate both irradiated tumors and tumors grown on distant sites.

Fe_3_O_4_ NPs have excellent biocompatibility, nontoxicity, MRI and magnetic targeting capability. Yang et al. demonstrated an R837 nanodrug carrier based on Fe_3_O_4_ superparticles, which can directly destroy tumors and activate the immune system by inducing DC maturation and secretion of cytokines with the help of NIR irradiation and an external magnetic field. The combination with anti-PD-L1 therapy can eradicate primary tumors directly exposed to PTT, prevent lung/liver metastasis, and inhibit the preexisting distant tumors after PTT (93). Recently, a kind of bone marrow mesenchymal stem cell (BMSCs) membrane-derived biomimetic nanovesicles attracts people’s attention (119). Researchers generate biogenic nanovesicles by utilizing low immunogenic BMSCs to express anti-PD-L1 antibodies and OVA antigen. Then, photosensitizer indocyanine green (ICG) and immunoadjuvants R837 were loaded into the nanovesicles by ultrasound. The anti-PD-L1 antibodies expressed on the nanovesicles can specifically bind to PD-L1 ligands on tumor cells and guide the nanovesicles home into tumor tissues. Under laser irradiation, the photosensitizer ICG mediated photothermal therapy efficiently ablates primary tumor and remodels tumor immune microenvironment. Meanwhile, immunoadjuvants R837 and OVA antigens stimulate DCs to activate the body’s immune response to residual tumors. In addition, Liu et al. developed a therapeutic strategy that coencapsulated indocyanine green (ICG) and R837 through oil-in-water emulsions to combine adjuvant nanoparticle-based PTT with ICB in colon cancer therapy (94).

Such strategies can offer stronger immunological memory effects to against tumors. R848 is another TLR-7 agonist. Li and his colleagues have designed using polydopamine (PDA) simultaneously loaded with R848 and carbon dots (CDs). The PTT effect of CDs triggered the release of R848, inducing a robust antitumor immune response. It can significantly potentiate the systemic therapeutic efficiency of PD-L1 therapy by activating both innate and adaptive immune systems in the body subsequently (95). Immunoadjuvant CpG can promote antigen presentation by DC maturation *via* binding to TLR-9 (89). You et al. coencapsulated hollow gold nanoshell and an anti-PD-1 peptide into nanoparticles. Their data demonstrated that perdurable PD-1 blocking combined with PTT and immune adjuvant could efficiently eradicate the primary cancer model CT26 tumor cells and inhibit the growth of metastatic tumors and their formation (92). The TLR5 agonist flagellin served as an excellent adjuvant to induce effective cell-mediated immunity. Rhee and his colleagues’ work investigated the effect of combining PDT and TLR5 agonist flagellin-adjuvanted tumor-specific peptide vaccination (FlaB-Vax) on promoting PD-1 blockade-mediated melanoma suppression. The combination of an immunoadjuvant with PDT effectively induced a systemic and local response of peptide tumor antigen-specific IFNγ secretion and the accumulation of effector memory CD8^+^ T cells, which further enhanced the PD-1 blockade therapeutic outcome in melanoma treatment (90).

### Phototherapy combined with other strategies to improve ICB therapy

In addition to targeted therapy, chemotherapy, and immune adjuvants, some antitumor strategies, such as anti-vascular therapy, starvation therapy, etc., can also synergize with photoimmunotherapy to inhibit tumor growth (96, 98). Anti-vascular medicine can break vessel wall barriers and change the TME to compensate for conventional phototherapy and immunotherapy limitations (120). CD276 is a receptor that is overexpressed in various tumor cells and tumor vasculature but with limited expression in normal tissues (121). Therefore, Liu and colleagues conjugated photosensitizer IRDye700 with the Fab fragment of an anti-CD276 antibody to combine antiangiogenic therapy and PDT with PD-1/PD-L1 blockade to treat primary tumors and ablation of tumor metastases. There was a marked increase in PD-L1 expression in 4T1 tumors after CD276 targeted PDT, which improved the efficacy of immune checkpoint inhibition (96).

Starvation therapy is also a good treatment option for cancer therapy. It can effectively inhibit tumor growth by cutting off the nutrition supply as a considerable supply of nutrients is needed for the rapid proliferation of cancer cells (122). Glucose oxidase (GOx) is an ideal endogenous natural enzyme for tumor starvation therapy (123). Lin and his colleagues constructed a multifunctional cascade bioreactor by starvation therapy/PTT/PDT/ICB therapy to treat cervical cancer. This bioreactor is based on hollow mesoporous Cu2MoS4 loaded with GOx for synergetic cancer therapy (98). In contrast, this quadri-combination therapy would more effectively release TAAs and elicit more robust immune responses. Further combining anti-CTLA4 antibody effectively eradicated both primary and metastatic tumors.

Melittin (MLT), the main component of bee venom, acts as a nonselective cytolytic peptide (124, 125). Yang et al. developed an organic-inorganic nanocarrier to load with Ce6 and MLT, denoted Ce6/MLT@SAB, aiming to simultaneously improve PDT-mediated intracellular ROS production and ICD levels. The addition of the anti-PD1 antibody further augmented antitumor effects, generating increased numbers of CD4^+^ and CD8^+^ T cells in tumors with concomitant reduction of myeloid-derived suppressor cells (97).

## The clinical application of phototherapy in cancer immunotherapy

Encouraging results from preclinical studies have prompted the clinical application of phototherapy in cancer immunotherapy. Phototherapy has been clinically or under clinical trials to treat solid tumors. Noninvasive phototherapy is very suitable for treating superficial cancers such as melanoma, osteosarcoma, squamous cell carcinoma, etc. Interstitial phototherapy, including laser interstitial thermal therapy (LITT) and interstitial photodynamic therapy (IPDT), can treat deep-seated tumors and avoid damage to healthy tissue by using the placement of interstitial laser fibers into tumors (126–128). Further development of LITT and IPDT allows broadening the scope of phototherapy application to treat tumors, such as lung cancer, esophageal cancer, prostate cancer, breast cancer, head and neck cancer, bile duct cancer, bladder cancer, pancreatic cancer, cervical cancer, brain cancer, etc. Phototherapy has considerable potential to be used in combination with ICB to treat a wide range of tumor types. Related clinical studies have shown that phototherapy combined with ICB therapy can reduce primary tumors, control untreated metastases, and prolong the survival of patients. This photoimmunotherapy has been used to treat patients with late-stage cancer who have failed other feasible treatment modalities.

### Lung cancers

Lung cancer remains the leading cause of cancer death in the latest global cancer statistics, with an estimated 18% of cancer-related deaths (129). Currently, phototherapy is increasingly being used to treat various forms of lung cancer, such as early-stage, advanced, or metastatic non-small cell lung cancer, multiple primary lung cancers, and small cell lung cancer (SCLC) (130). FDA approved Porfimer sodium (Photofrin) for the treatment of palliation of patients with esophageal cancer, treatment of microinvasive endobronchial non-small-cell lung cancer, and reduction of obstruction and palliation of symptoms in patients with wholly or partially obstructing endobronchial non-small cell lung cancer (131). Talaporfin is a second-generation photosensitizer approved in Japan (132). It can be clinically used in PDT for early-stage lung cancer, primary malignant brain tumors, and locally remnant recurrent esophageal cancer. Phototherapies are also attractive options for treating malignant pleural mesothelioma (MPM) (133).

In April 2021, a phase I clinical trial was initiated at Roswell Park Cancer Institute to evaluate PDT’s ability to amplify the response to immunotherapy in patients with non-small cell lung cancer with pleural disease (NCT number: NCT04836429). Sixteen patients are expected to be enrolled to receive porfimer sodium IV over 3-5 minutes 24-48 hours prior to standard of care VATS. This trial evaluates the side effects of intraoperative PDT with porfimer sodium in enhancing the response to an ICI drug. The incidence of serious adverse events (SAEs) was determined by recording the occurrence of SAEs during the first 28 days of poststudy-related immunotherapy. In addition, patients were followed for progression-free survival (PFS), overall survival (OS), changes in the immune phenotype of peripheral blood CD8^+^ T cells, and changes in the platelet-to-lymphocyte ratio within a time frame of two years. The study is expected to be completed in December 2023 (**Table 2**).

**Table 2:** Clinical trials of combination of phototherapy and ICB therapy in malignant tumors.

Clinical trail	Phase	Year	Cancer type	Phototherapy	Phototherapy agent	ICB	ICB agent
NCT04836429	Phase I	2021	Non-small cell lung cancer with pleural disease	PDT	Porfimer sodium	PD-1	ICIs
Case Report (134)	Clinical Case Report	2018	Head and neck squamous cell carcinoma (HNSCC)	PDT	Redaporfin	PD-1	Nivolumab
NCT03727061	Phase I/2	2018	Head and Neck Cancer	PDT	Porfimer sodium	PD-1	Nivolumab/Pembrolizumab
NCT04305795	Phase I/2	2020	Head and Neck Cancer	PIT	ASP-1929	PD-1	Pembrolizumab/Cemiplimab
NCT05220748	Phase I	2022	Head and Neck Cancer	PIT	ASP-1929	PD-1	Pembrolizumab
NCT05265013	Phase II	2022	Head and Neck Cancer	PIT	ASP-1929	PD-1	Pembrolizumab
Case Report (135)	Clinical Case Report	2017	Melanoma	LIT	Laser	CTLA-4	Ipilimumab

### Head and neck cancer

Head and neck cancer is the seventh most common type of cancer worldwide. It comprises a diverse group of tumors affecting the upper aerodigestive tract (136). Several multi-institutional phase II clinical trials of PDT have demonstrated its efficacy in treating early oropharyngeal primary and recurrent cancers and the palliative treatment of refractory head and neck cancers. Patients with early-stage cancers or early recurrences in the oral cavity and larynx respond well to PDT (137). In July 2015, a phase I clinical trial was initiated to evaluate the safety and antitumor activity of RM-1929 in patients with terminal head-and-neck cancer (NCT number: NCT02422979). RM-1929 is a chemical conjugate of the dye IR700 with an EGFR receptor-targeting antibody. Composed of a silica core and a gold shell, “AuroShell” NPs have also been used for pilot clinical trials in PTT applications to treat head and neck cancer (NCT00848042) (**Table 2**).

A successful treatment option combined the ICI nivolumab with redaporfin-mediated PDT to treat a patient with head and neck squamous cell carcinoma (HNSCC) in 2018. The patient had failed numerous prior therapies, including surgery, radiotherapy, and multiple lines of systemic treatment. PDT destructed all visible tumors with Redaporfin, and the combination of an ICI immunotherapy promoted a complete sustained response (134) (**Table 2**).

In addition, several early-phase clinical trials combining phototherapy with ICB have been initiated. A phase 1/2 (NCT number: NCT03727061) study of 82 patients was launched in 2018 to study how I-PDT works with standard care of cetuximab, nivolumab, or pembrolizumab. The study is expected to be completed in July 2023. Asp-1929 is also a photoimmunotherapy agent with a similar structure to RM-1929, and its current clinical trials are focused on head and neck cancer. In 74 patients with recurrent or metastatic head and neck and squamous cell cancer or advanced or metastatic cutaneous squamous cell carcinoma, an open-label study using ASP-1929 photoimmunotherapy combined with anti-PD1 therapy was launched in 2020 (NCT number: NCT04305795) (**Table 2**). The estimated study completion date for this trial is June 2024. In addition, two clinical studies started this year. One is to use RM-1995 with pembrolizumab in 36 patients with advanced cutaneous squamous cell carcinoma (cuSCC) or head and neck squamous cell carcinoma (HNSCC) to evaluate the safety and efficacy (NCT number: NCT05220748). Another study combined ASP-1929 with pembrolizumab in 33 patients with locoregional recurrent squamous cell carcinoma of the head and neck, with or without metastases (NCT number: NCT05265013) (**Table 2**).

### Skin cancer

Skin cancers are generally classified into melanoma skin cancer (MSC) and non-melanoma skin cancer (NMSC) (138). Laser immunotherapy (LIT) is a promising modality that combines local, selective PTT with immunological therapy to treat metastatic melanoma (139). Non-melanoma skin cancer or precancerous cutaneous lesions, including basal cell and squamous cell skin cancers and actinic keratosis, are essential indications for PDT. Tens of millions of patients are likely to have been treated by PTT/PDT worldwide to date (140, 141).

LIT induces long-term antitumor immunity by enhancing antigen uptake and presentation, leading to an enhanced response to ICIs such as ipilimumab. In one exciting example, one patient with advanced (stage IV) melanoma used the combination of LIT and ipilimumab. The patient received treatment with imiquimod and an 805 nm diode laser on the target sites for three months. After three months, all treated cutaneous melanomas in the head and neck cleared completely. Then the patient was treated with ipilimumab. After ipilimumab treatment, all lung tumor nodules entirely resolved (**Table 2**) (135).

### Esophageal cancer

Esophagectomy carries a high risk of postoperative complications and mortality. Esophageal cancer was responsible for one in every 18 cancer deaths in 2020 global cancer statistics (129). Thus, PDT is considerably appealing as a locally minimally invasive treatment. PDT has been approved as a curative treatment for superficial esophageal squamous cell carcinoma (ESCC) in Japan and approved for dysplastic Barrett’s esophagus and palliative treatment for symptomatic obstructive esophageal cancer in the US (142). In recent years, PDT has regained popularity due to the invention of second-generation PDT using talaporfin sodium and diode laser. The efficacy and safety of PDT as a salvage treatment for patients with local failure after chemoradiotherapy (CRT) have been demonstrated in several clinical trials (143).

### Prostate cancer

Prostate cancer was the second most frequent cancer in 2020. There are primarily four photosensitizers, including Tibofen, Motexafin Lutetium (MLU), Vitibofen, and Padeliporfin, used in clinical PDT for prostate cancer (144). PDT using various photosensitizers for the focal ablation of prostate tumors has been tested in clinic (145). Recently, AuroShells, which are tiny silica spheres with a thin outer shell of gold, were developed for PTT treatment of patients with prostate cancer in a clinical pilot study. Fifteen men aged 50-79 years with low to medium-risk localized prostate cancer were treated with PTT. There were no detectable signs of cancer in 86.7% (13/15) of patients within one year (146). “AuroShell” NPs have been used in pilot clinical trials to treat men with low- or intermediate-risk prostate cancer (NCT04240639).

### Breast cancer

Female breast cancer has now surpassed lung cancer as the leading cause of global cancer incidence, representing 11.7% of cancer cases (129). In recent years, phototherapy has made significant progress in breast cancer research. LITT has been explored to treat benign breast tumors (NCT00807924). A multicenter clinical trial was designed to determine the efficacy and outcome of percutaneous laser ablation (PLA) in treating invasive ductal breast carcinoma (IDC). In this trial, 51 (84%) of 61 patients had complete tumor ablation confirmed by pathology analysis (147). In a clinical trial in Peru, ten breast cancer (stage III or stage IV) patients considered out of other available treatment options were enrolled in a photothermal immunotherapy clinical trial, using ICG as the photo agent and GC immunoadjuvant. In 8 patients available for evaluation, the overall 3-year survival was significant. These patients only had a life expectancy of 3-6 months prior to the study (148).

### Other indications

In addition to the indications mentioned above, PDT has also been tested clinically for dozens of other cancer indications, including bladder cancer (149), brain cancer (150, 151), cholangiocarcinoma (152, 153), pancreatic cancer (154), and gynecological cancers (155). Bladder cancer was the first indication for which porfimer sodium was approved in Canada in 1993. TLD-1433 is the first ruthenium (II)-based photosensitizer for photosensitizer to enter human clinical PDT trials to treat nonmuscle invasive bladder cancer. (NCT03945162). The FDA approved oral ALA for the fluorescence-guided resection (FGR) of high-grade gliomas in 2017 (150).

## Conclusions

There is growing evidence that nanotechnology-based phototherapy can improve ICB treatment efficacy by regulating the anti-tumor immune response. In addition to mediating PTT and PDT for cancer therapy, photosensitive nanocarriers also serve as drug carriers to deliver small molecular inhibitors, chemotherapeutic drugs, or immunomodulators into tumor tissues to treat tumors or regulate tumor microenvironment. Therefore, combing ICBs with nanotechnology-based phototherapy is one of the most promising strategies in anticancer therapy.

In PTT/PDT, the photothermal/photosensitizer agents convert the light energy to heat and elevate the temperature of tumors to the cytotoxic level or generate cytotoxic ROS to ablate tumor cells. There is no doubt that nanoparticle-based PTT/PDT combined with ICB therapy has produced impressive preclinical results. However, most of these studies are still at the laboratory stage and face multiple challenges that limit their full synergistic potential with cancer immunotherapy. One major challenge is the limited penetration depth of red and NIR light for deep-seated tumors. As a result, phototherapy is commonly used in the treating specific skin carcinoma and superficial cancers. To solve this problem, interstitial and NIR-II phototherapy were developed to circumvent these barriers. Recently, MR-guided LITT, as a minimally invasive treatment modality, has been used to treat primary and metastatic brain tumors (156, 157). In addition, light in NIR-II has a higher upper limit of radiation and greater tissue tolerance than in the NIR-I window. NIR-II treatment can trigger more homogeneous and deeper immunogenic cancer cell death in solid tumors (158). In recent, a NIR-II SWNT modified by a novel immunoadjuvant, glycated chitosan (GC), was used by Chen and colleagues to treat metastatic mammary tumors in mice. After intratumoral administration of SWNT-GC, they used a 1064 nm laser to irradiate the primary tumors to achieve local ablation through PTT. Combination with anti-CTLA-4 antibody produced systemic antitumor immunity that inhibited lung metastasis and prolonged the survival time of the treated animals (159). We believe that with more research into this field, the current library of phototherapy agents will certainly be expanded and a “blockbuster” agent to solve this problem is just around the corner. It is also worth mentioning that the FDA has published particular guidance for nanomedicines until recently, as they are categorized under complex products with multiple components (160, 161). Although nanoparticles of phototherapy are typically better agents, caution should be exercised when selecting them, as there are some questions still remain on their safety and toxicity profiles. Consequently, it is necessary to develop the targeting ability of phototherapy agents to increase the accumulation of drugs in tumor tissue or residual tumor cells. The current targeting strategies use high-affinity ligands or targeting moieties on the surface of the nanocarriers that bind to specific overexpressed receptors on the target cancer cells (162). Standard ligands or targeting moieties include antibodies or antibody fragments, aptamers, carbohydrates, human transferrin protein, peptides, and vitamins such as folate (163, 164).

Improving photothermal conversion efficiency (PCE) is essential to facilitate therapeutic performance during PTT should be considered as well. An ideal photothermal agent would have a high PCE without absorption disturbance from the chromophores in biological tissue. Photothermal agents with high PCE have been developed with the advancement of PTT research (165–167). In recent years, many efforts have been made to improve PCE (168). Reducing the fluorescence emission and inhibiting the singlet to triplet state intersystem crossing (ISC) reaction are valuable strategies for improving heat generation (169). In addition, reducing tumor heat tolerance is another strategy to address this problem. For example, the tolerated thermal ranges were reduced in hypoxia, suggesting that the combination of PDT and PTT may be a good choice (170). Lastly, strong oxygen dependence in PDT is another main challenge in ablating tumor cells. Rapid tumor growth and insufficient blood supply lead to a hypoxic microenvironment in tumors (171). Moreover, local oxygen consumption of PDT also aggravates tumor hypoxia, which can seriously affect the efficacy of PDT. Multifunctional delivery strategies have been proposed to overcome this problem, such as oxygen-replenishing strategies (using oxygen carriers and generators to deliver oxygen into tumors), downregulation of oxygen consumption, and O_2_-independent strategies (subcellular organelle-targeted and O_2_-independent PDT), and hypoxia utilization (combinations of hypoxia-responsive chemotherapeutic drugs) (172–174).

Combining ICB with the assistance of phototherapy could greatly improve cancer treatment effects as described in this review. With the development of biomedical and optical technologies, reduction of costs, and in-depth study of biological mechanisms, more novel, and innovative photosensitive agents will continue to be developed to solve the above problems. Phototherapy combined with ICB therapy is under investigation in large-cohort clinical trials and has the potential to move forward as next-generation technology for cancer treatment. The path of clinical transformation of the combination therapy strategy of “PTT/PDT + ICB” has begun.

## Author contributions

All authors listed have made a substantial, direct, and intellectual contribution to the work and approved it for publication.

## Funding

This work was funded by the National Natural Science Foundation of China (No. 22105137 and No. 82172634); Key Program of the Science and Technology Bureau of Sichuan (No. 2021YFSY0007); 1.3.5 project for disciplines of excellence, West China Hospital, Sichuan University (No. ZYYC20013; China Postdoctoral Science Foundation (No. 2020M683324); Post-Doctor Research Project, West China Hospital, Sichuan University (No. 2020HXBH051).

## Conflict of interest

The authors declare that the research was conducted in the absence of any commercial or financial relationships that could be construed as a potential conflict of interest.

## Publisher’s note

All claims expressed in this article are solely those of the authors and do not necessarily represent those of their affiliated organizations, or those of the publisher, the editors and the reviewers. Any product that may be evaluated in this article, or claim that may be made by its manufacturer, is not guaranteed or endorsed by the publisher.
